# Magnetic nanowires substrate increases adipose-derived mesenchymal cells osteogenesis

**DOI:** 10.1038/s41598-022-21145-z

**Published:** 2022-10-06

**Authors:** Luminita Labusca, Camelia Danceanu, Anca Emanuela Minuti, Dumitru-Daniel Herea, Adrian Ghemes, Cristian Rotarescu, Oana Dragos-Pinzaru, Mihai Tibu, Grigoras Marian, Horia Chiriac, Nicoleta Lupu

**Affiliations:** 1 Department of Magnetic Devices and Materials, National Institute of Research and Development for Technical Physics, 700050 Iasi, Romania; 2 Orthopedics and Traumatology Clinic, County Emergency Hospital Saint Spiridon Iasi, 700111 Iasi, Romania; 3 Alexandru Ioan Cuza University, Faculty of Physics, 700506 Iasi, Romania

**Keywords:** Biotechnology, Cell biology, Stem cells, Medical research, Materials science, Nanoscience and technology, Physics

## Abstract

Magnetic nanomaterials are increasingly impacting the field of biology and medicine. Their versatility in terms of shape, structure, composition, coating, and magnetic responsivity make them attractive for drug delivery, cell targeting and imaging. Adipose derived-mesenchymal cells (ASCs) are intensely scrutinized for tissue engineering and regenerative medicine. However, differentiation into musculoskeletal lineages can be challenging. In this paper, we show that uncoated nickel nanowires (Ni NW) partially released from their alumina membrane offer a mechanically-responsive substrate with regular topography that can be used for the delivery of magneto-mechanical stimulation. We have used a tailored protocol for improving ASCs adherence to the substrate, and showed that cells retain their characteristic fibroblastic appearance, cytoskeletal fiber distribution and good viability. We report here for the first time significant increase in osteogenic but not adipogenic differentiation of ASCs on Ni NW exposed to 4 mT magnetic field compared to non-exposed. Moreover, magnetic actuation is shown to induce ASCs osteogenesis but not adipogenesis in the absence of external biochemical cues. While these findings need to be verified in vivo, the use of Ni NW substrate for inducing osteogenesis in the absence of specific differentiation factors is attractive for bone engineering. Implant coating with similar surfaces for orthopedic and dentistry could be as well envisaged as a modality to improve osteointegration.

## Introduction

Magnetic nanomaterials are increasingly tested for regenerative medicine (RM) purposes. The combined nano-scale dimensions and magnetic properties are recommending them as versatile platforms for various applications in drug delivery, cell sorting, targeting and tracking of cell populations or subcellular components^1^. Recently, mechanical stimulation mediated by magnetic nanoparticle (MNP) actuation within magnetic field (MF) has been used to manipulate mesenchymal stem cell fate especially for musculoskeletal lineage differentiation: bone^2,3^, tendon^4^ or cartilage^5,6^. Indeed, mechanical stimulation of mesenchymal stem cells (MSC) of various origins was shown to increases differentiation to musculoskeletal lineages (bone, cartilage, and skeletal muscle) both in vitro and in vivo^7^. However, the modality of providing the appropriate micro-mechanical stimulation that closely mimic the intensity, duration and orientation of mechanical cues within the developmental niche, has proved to be a challenge for musculoskeletal tissue engineering. Adult stem cells and especially MSC are known to respond to topographical and micro-topographical cues of the substrate they adhere on^8^. Substrate geometry and stiffness is known to orient MSC differentiation, being important for guiding tissue engineering strategies. Soft hydrogels were shown to support MSC proliferation as well as adipose and neural differentiation^9,10^ while stiff and elastic substrate favor osteogenesis and chondrogenesis, respectively. Stem cells respond to intracellular and extracellular mechanical stimuli through changes in cytoskeletal dynamics, actin and microtubule re-polymerization and consequent changes in the nuclear membrane with a role in initiating conversion to a particular cell line. For this reason, MSC mechanic-responsivity can be used to direct differentiation to the desired cell lineage for tissue engineering purposes^11^. Magnetic nanowires (NW) can be used for their dual effect in altering the topography of the adhesion substrate and in providing magneto-mechanical stimulation during the initiation of differentiation processes. It has been shown that mesenchymal stem cells can be cultured on iron nanowire substrates, with cells changing their cytoskeletal orientation and adopting a spherical morphology compared to their elongated shape displayed on regular tissue culture dish^12^ probably promoting adipogenic conversion. Moreover, bone marrow MSC cultured on magnetic NW within a magnetic field were shown to promote osteogenic differentiation^13^. Adipose-derived mesenchymal stem cells (ASCs) are intensively tested in preclinical and clinical studies for their use as cell sources in RM^14^. ASCs are relatively easy to extract by enzymatic or mechanical methods from adipose tissue removed during elective cosmetic procedures. ASCs are available as both autologous and allogeneic sources, can derive larger number of elements per tissue volume and display increased proliferative and immunomodulatory capabilities compared with MSC of other origins^15^. However, ASCs conversion to musculoskeletal lineages, especially to hard tissues, has proved to be less efficient compared to other MSC sources, impeding their use in engineering of musculoskeletal hard tissues such as bone, cartilage or tendon. The delivery of appropriate micro mechanical stimulation that mimics the intensity, duration and orientation of mechanical cues in the developmental niche, at cellular level, has proven not trivial. The use of sophisticated bulky equipment for the delivery of mechanical stimulation consistently complicates the procedure of obtaining implantable engineered musculoskeletal bioequivalents.

Here, soft magnetic parallel-aligned, tightly-packed Ni NW were investigated as a substrate able to deliver discrete micromechanical stimulation to differentiating ASCs in vitro. Taking advantage of the mechanical responsiveness of NW substrates, we sought to test the feasibility of improving ASCs osteogenesis, delivering magneto-mechanical stimulation by means of intermittent exposure to alternating magnetic field (MF). Theoretical modelling of magneto-mechanical actuation was performed to approximate the MF distribution around NW endings. Human primary ASCs cultured on NW substrate were tested for the acquired cell morphology, viability, cytoskeletal fibers appearance, as well as for the ability to undergo adipogenic and osteogenic conversion in determined culture condition. Osteogenic and adipogenic conversion in ASCs cultured on soft Ni NW exposed and non-exposed to MF was quantified and compared to differentiation on regular plastic surfaces in similar conditions.

## Results

### NW platforms characterization

**Figure 1:**
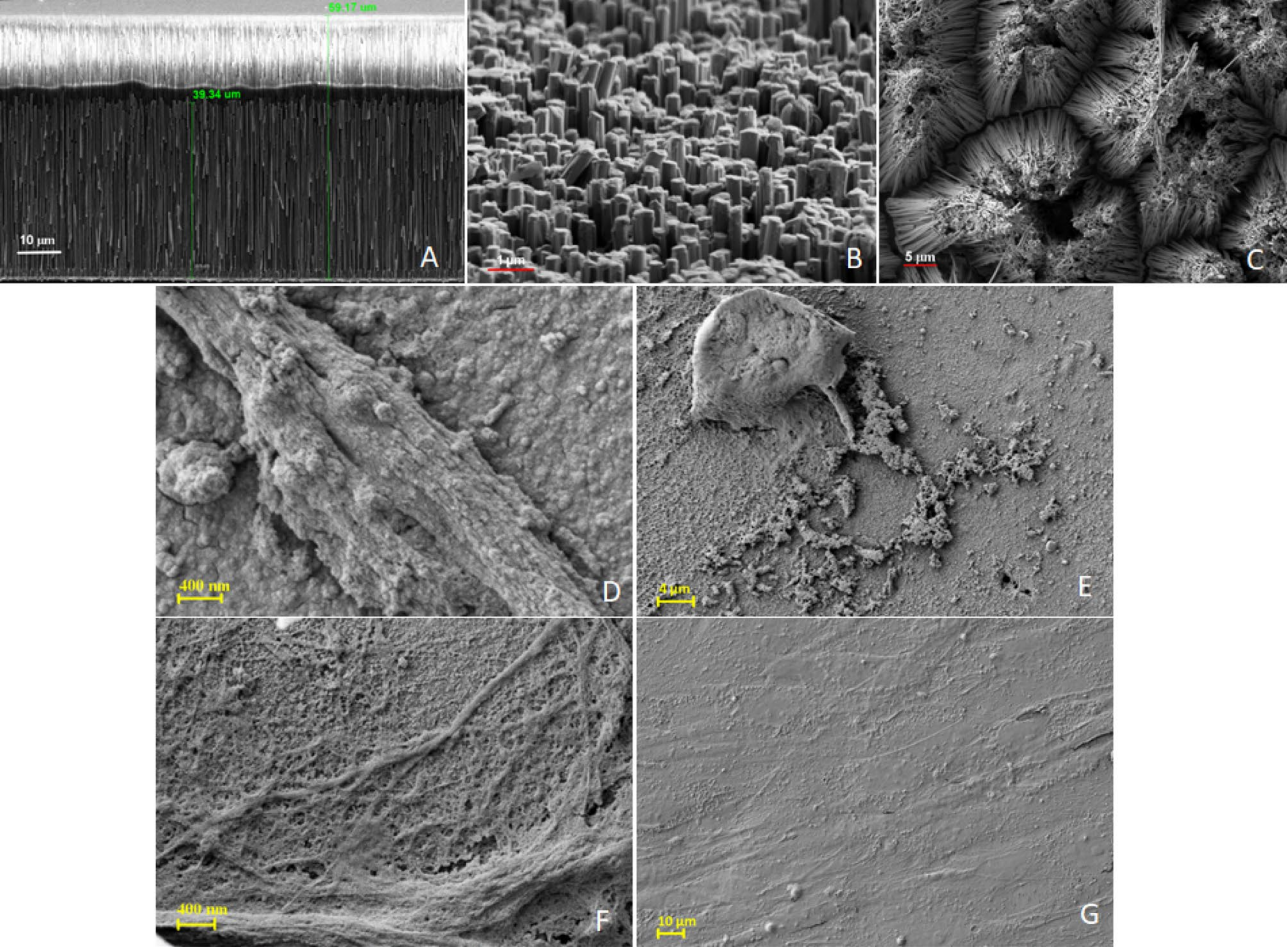
Scanning electron microscope (SEM) images of Ni NW: (**A**) 40 μm long NW deposed in a membrane of about 60 μm thickness; (**B**) surface of the partial dissolved membrane showing irregular NW tips; (**C**) groups of nanowires tips after dissolving completely the alumina membrane: ADSC on NW substrates (**D**,**E**) ADSC on NW placed in adherent cell culture plates are rounded or tubular with ruffled membranes; (**F**,**G**) ADSC cultured in ultralow adherent plates (ULA plates). Cells maintain characteristic fibroblastic morphology without apparent breach of cellular membrane however lower density and ruffled membrane features can be observed in ADSCs on adherent plates.

The physical characterization of the nanowires was performed by SEM. As Fig. 1A shows, Ni NW were about 40 μm long. By treating the free top surface of the alumina membrane with NaOH solution, part of the membrane was dissolved, exposing the nanowires over a length of a 1–2 μm (Fig. 1A). The exposed ends are generally parallel to each other after chemical treatment while part of them is in contact (Fig. 1B). Also, the tips are not regularly distributed, providing cells a rough adherent surface which may be a more suitable approach to strongly immobilize the cells. If released from alumina membrane, the Ni NW have a strong tendency to group in small bunches, especially towards the ends (Fig. 1C). Therefore, the dissolving process must be well controlled to allow for a firm anchoring structure based on slightly flexible nanowire tips to keep the plasma membrane of the cells unaffected during the application of alternating magnetic fields.

### Magnetic characterization

**Figure 2:**
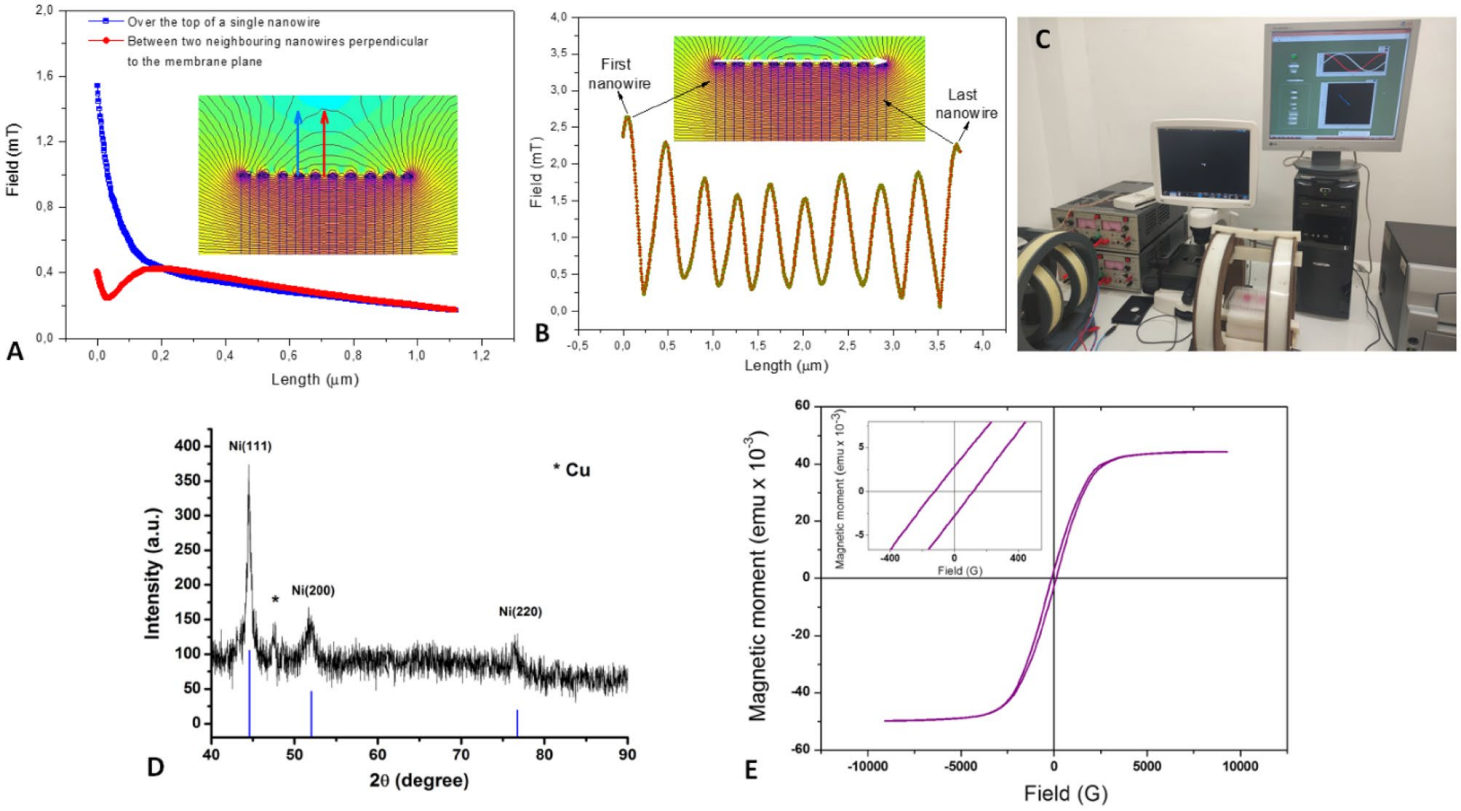
Theoretical simulation of NW magneto mechanical effect. (**A**) Magnetic field variation on vertical direction above one wire (blue arrow) and between two nanowires (red arrow), respectively; (**B**) magnetic field variation on horizontal direction (white arrow) over the tips insets of (**A**) and (**B**): magnetic nanowires subjected to magnetic field produced by the Helmholtz coil simulated using FEMM software; (**C**) experimental setup comprising the Helmholtz coils, source of current, and computer with the proprietary software for coil control; (**D**) XRD patterns of Ni NW; (**E**) magnetization loop of the NW (inset: details from the region of near zero magnetic field).

For VSM, the applied magnetic field was perpendicular to the long axis of the nanowires. In this configuration, the magnetization loop of the nanowires-filled alumina membrane showed a typical ferromagnetic behavior, with a coercive field of 118 G and a remanent field of about 5% from the saturation magnetization which explain the tendency for agglomeration of the Ni NW observed in Fig. 2E.

### XRD analysis

The XRD patterns of NW pointed out specific diffraction peaks of nickel, corresponding to (110), (200) and (220) diffraction planes, along with a copper specific peak coming from the copper substrate on which the nanowires were electrochemically deposited (Fig. 2D).

### Theoretical simulation

The theoretical model used a random distribution of the nanowires’ heights to reproduce to some extent the experimental conditions, given that the heights of the nanowires in the alumina membrane differ slightly.

As shown in the insets of Fig. 2A,B, high-density magnetic nanowires in the membrane strongly distort and concentrate the homogeneous magnetic field flowing perpendicular to the long axis of the nanowires and parallel to the membrane surface. The magnetic field is most intense around the tips of the nanowires, inside the NW-filled membrane and on the edges, while less intense between nanowires’ tips.

Based on the graphs in Fig. 2A,B, we evaluated the field gradient above the end of a nanowire (i.e. the fourth—blue arrow in Fig. 2A). Gradient values of approximately 7 mT/µm were found for the first 0.15 µm, and 0.3 mT/µm for the next 0.97 µm above the tip of the nanowire. When the analysis focused on the magnetic field variation in the region starting with the middle distance between two neighboring tips (nanowires 5 and 6—red arrow in Fig. 1B), for 0.15 µm distance above nanowires, an initial variation of 0.16 mT (field gradient ≈ 5 mT/µm), followed by an increase of 0.17 mT (field gradient—1.5 mT/µm) was obtained. Then, the curves overlap, following a slow, almost linear decrease. MF intensity follows a sinusoidal distribution (Fig. 2B) over NW tips when analyzed linearly, and the profile of a spherical/conical cover, respectively, if analyzed three-dimensionally.

For ideal conditions, single nanowire, and assumed small deflections of the ends (as possible in our experimental approach due to the very short tips), produced under the action of the AMF, the radius of curvature of the nanowire is expressed as^16^:

$${R}_{C}= \frac{{\mu }_{0}E{D}^{2}}{8\Delta \mathrm{\chi Lsin}\left(2\beta \right)}\frac{1}{{B}^{2}}$$

where, *µ*_*0*_ is the magnetic permeability, *E*—Young modulus, *D*—diameter of the nanowire, *χ*—susceptibility, *L*—the length of the nanowire, β—angle between the initial nanowire orientation and that of the applied field, *B*—the applied magnetic field.

For longer ends, larger deflections occur, which add a number of additional forces to the magnetic and elastic, along with the complexity introduced by the nanowire-nanowire magnetic/electrostatic interaction, such that the calculation of the radius of curvature and the minimum intensity of the magnetic field, for which the nanowires’ tips can still bend, becomes very complex.

### ASCs adherence, morphology and cytoskeleton appearance on NW substrate

We tested the ability of ASCs to adhere to NW-filled alumina membrane (NW substrate) by seeding ASCs cell suspension in adherent and ultra-low adherent (ULA) plates. SEM images ascertained that in both culture conditions, ASCs adhere on NW substrates. Characteristic mesenchymal cell morphology is retained in the case of ULA plates with fusiform—fibroblastic appearance of cellular body and single round nuclei, without any apparent breach of cellular membrane; by contrary, with increased membrane ruffing and a rounded cell shape in the case of NW substrates in adherent plates. When placed within adherent culture surface, ASCs tend to attach solely on the edges of alumina membrane, while a uniform distribution of cells on membrane is obtained for ULA plates (see below).

SEM images revealed that very few cells placed in adherent plates are retained on NW substrates, making it difficult to detect their presence. Few cellular elements displaying rounded cell shape or, alternatively, elongated with ruffled membrane could be observed (Fig. 1D,E). Conversely, cells on NW substrate placed within ULA plates can be found in larger numbers. Cells retain their fibroblastic-like appearance with elongated cellular bodies and smooth membrane appearance (Fig. 1F,G) spreading all over NW substrate.

**Figure 3:**
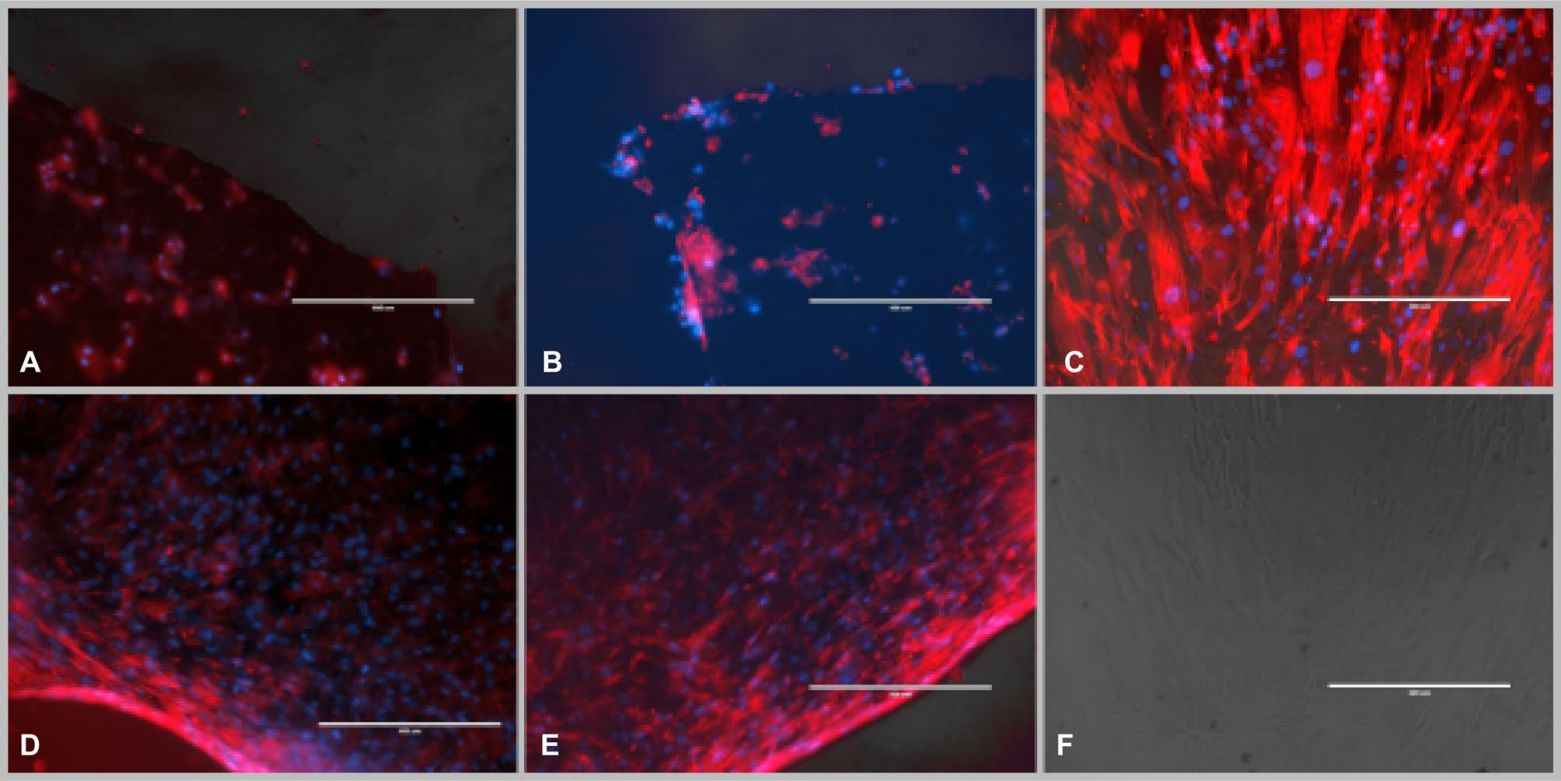
ASCs F-actin fibbers on Ni NW: (**A**,**B**) ADSC on NW placed in adherent culture plates; few visible cell bodies with rounded shape and predominant marginal distribution; (**C**) ASCs cultured on regular adherent culture plate demonstrating their fibroblastic like morphology; (**D**,**E**), ASCs on NW placed in ultra-low adherent culture plates (ULA) cells occupy entire NW surface and form intermingled cellular networks; increased number of cells demonstrated by the density of cellular nuclei; (**F**) ASCs on regular culture substrates, transmission optic electron microscope. In red- F actin fibers stained with phalloidin (Texas Red) in blue cell nuclei stained with DAPI.

Cell cytoskeleton appearance was qualitatively investigated using Phalloidin Texas red staining – a red fluorescent dye that interact with F actin fibers within cytoskeletal cell apparatus. In ASCs cultured on NW in adherent plates, cytoskeleton fibers where very much shortened and rounded, suggesting a modified cell shape that tends not to spread on the substrate but rather retains a round configuration and do not interconnect to other cellular elements (Fig. 3A,B). For ASCs cultured on ULA plates, however, ASCs retain characteristic fibrillary shape with long parallel bundles that tend to agglomerate (Fig. 3D,E), cellular bodies forming intertwined networks. This suggest a cell shape and cytoskeleton structure that is closer to typical fibroblast-like appearance of ASCs plated in regular adherent culture dish (Fig. 3C,F).

### ASCs viability on NW substrate

**Figure 4:**
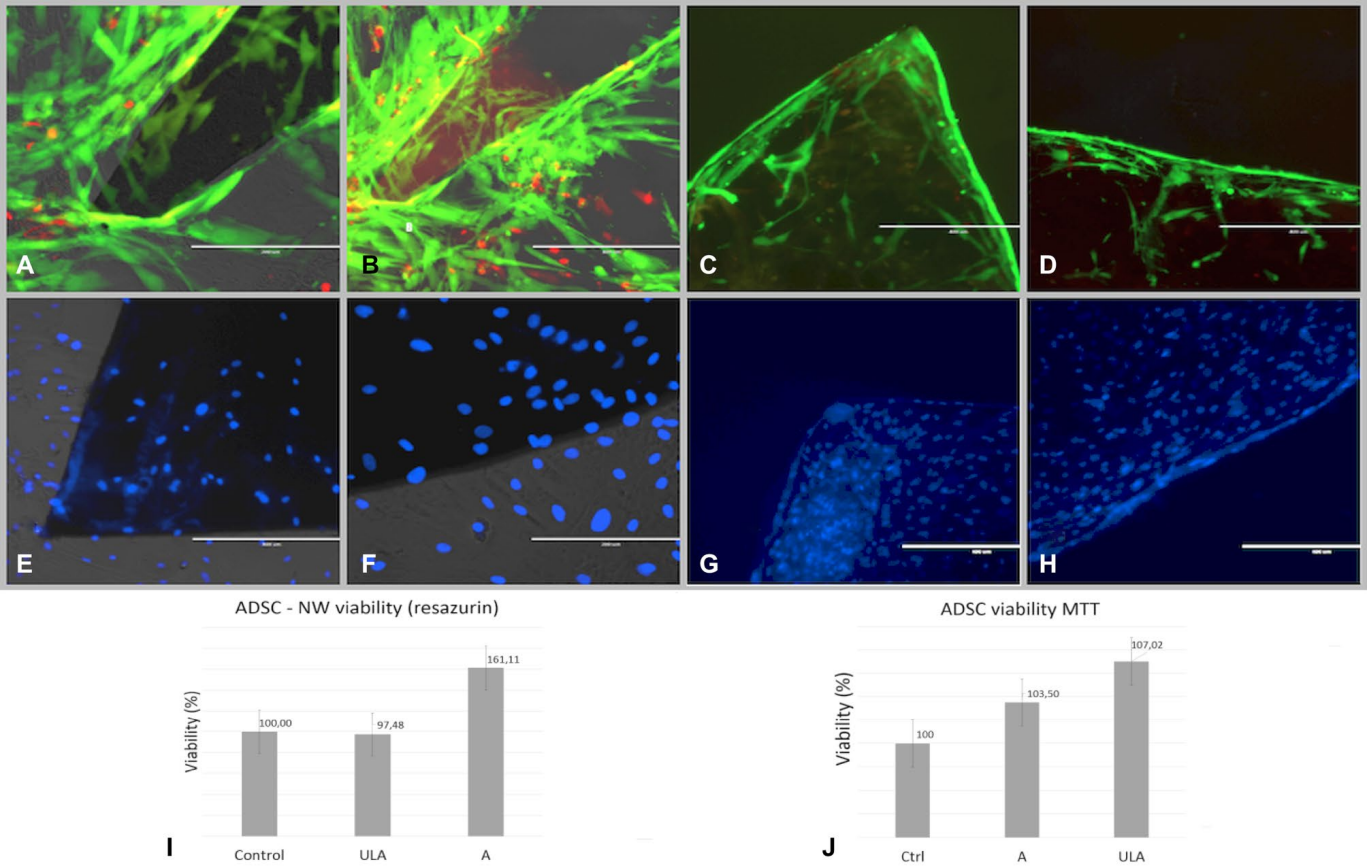
ASCs cultured on nanowire as substrate: in adherent plates (**A**,**B**,**E**,**F**); (**A**,**B**) life dead staining of ASCs cultured on nanowire substrate after 48 h, good cell viability—in green fluorescent calcein staining of viable cells in red—ethidium bromide fluorescent staining of non-viable cells; (**E**), (**F**) nuclear staining with DAPI demonstrating cells are capable to adhere on both plastic culture dish surface as well as on nanowire substrate: in non-adherent plates (**C**,**D**,**G**,**H**); (**C**,**D**) life dead staining of ASCs cultured on NW substrate after 48 h demonstrating good cell viability with very few red non-viable elements; (**G**,**H**), nuclear staining demonstrate cells adhere on NW but not on cell culture plastic surface; (**I**) ASCs viability on Nanowire substrate assessed with Resazurin assay; (**J**) ASCs viability assessed with MTT assay; A = Adherent plates ULA = ultralow adherent plates NW = nanowire substrate.

Cell viability was tested quantitatively using colorimetric tetrazolium salt—based MTT assay as well as with resazurin (7-hydroxy-3H-phenoxazin-3-one-10-oxide), also referred to as alamar Blue or Presto Blue assay. Both tests are designed for investigation of mitochondrial metabolic activity and mainly of availability of NADH and NADPH as electron sources. Viable cells are able to induce colorimetric changes of the specific dyes that results in a quantifiable color change. ASCs viability at 72 h after plating was found to be comparable to cells cultured on NW substrate for both adherent and ULA plates. Slight differences, non-statistically significant, were found to be assay-dependent, with MTT revealing increased viability on cells deposited on NW substrate placed in ULA plates compared to normal culture conditions, and resazurin displaying slight increased viability on ASCs on NW in adherent plates (Fig. 4I,J). The fact that ASCs retain comparable viability on NW substrate placed in both adherent and ULA plates was qualitatively confirmed by the LIVE/DEAD assay that detects cell membrane integrity as well as cell esterase activity. The green, fluorescent dye calcein is retained by living cells that appear green in fluorescence microscopy while red fluorescent ethidium bromide is excluded due to intact esterase activity. In dying, membrane impaired or esterase deficient cells, ethidium bromide is retained; therefore, cells are visible as red spots in fluorescent microscopy. We could detect predominantly green ASCs cellular bodies attaching solely on NW substrate in the case of ULA plates (Fig. 4C,D) while cells were spread all over culture dish and predominantly at the edges in the case of NW placed within adherent culture dish (Fig. 4A,B). The presence of cells solely on NW (Fig. 4G,H) and not in the rest of the culture dish for the case of ULA plates was further confirmed by nuclear staining with DAPI (Fig. 4E–H).

Given the good viability and preservation of cell shape observed with qualitative and quantitative viability tests as well as by means of imaging of cytoskeletal actin fiber arrangement, further experiments involving differentiation assays were performed using solely using NW placed within ULA plates.

### ASCs differentiation on NW substrate—adipogenesis

**Figure 5:**
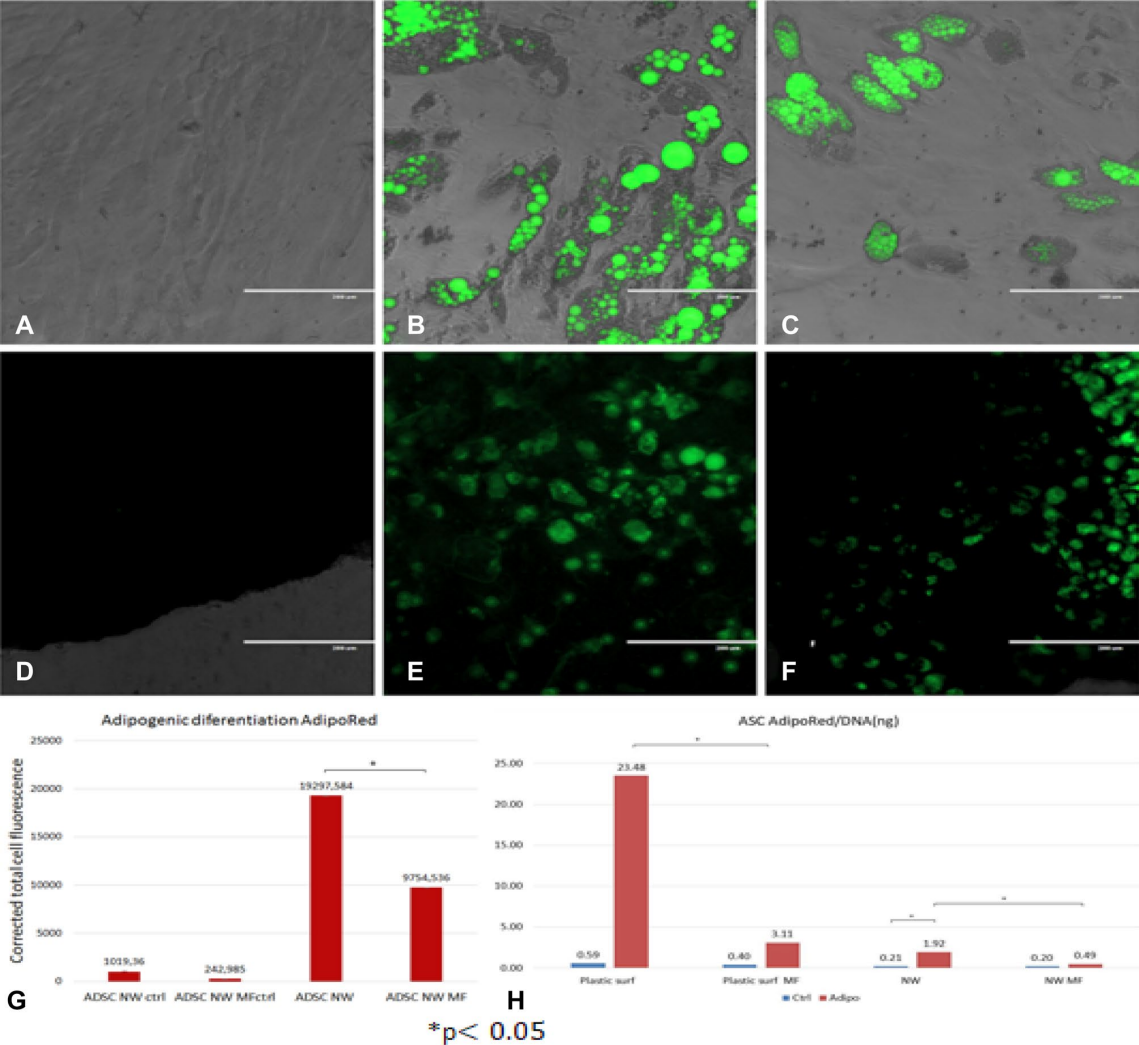
ASCs differentiation: (**A**–**F**) fluorescence microscope images of adipogenic differentiation: (**A**) control (ASCs on regular adherent culture plates in simple culture media); (**B**) ASCs in regular lates in adipogenic media; (**C**) MF exposed ASCs in adipogenic media; (**D**) ASCs on NW substrate in simple culture media; (**E**) ASCs on NW substrate in adipogenic media; (**F**) ASCs on NW substrate exposed to MF in adipogenic media; AdipoRed^®^ fluorescent staining of intracellular lipid accumulation, all images represent overlay of transmitted and GFP fluorescence images captured with inverted fluorescent microscope (EVOS Life Sciences); (**G**) semiquantitative assessment of AdipoRed^®^ fluorescence using Image J software; (**H**) Quantitative assessment of AdipoRed^®^ fluorescence using spectrophotometric plate reader relative to DNA content.

ASCs were exposed to differentiation media 48 h after seeding on NW substrate placed within ULA plates. Differentiation assays were performed in parallel on ASCs in normal culture plates as controls. MF exposure was performed by introducing differentiation assays within the Helmholtz coils. MF exposure was performed for 10 min every 2 h, in the first 7 days of adipogenic media exposure. After 21 days, qualitative assessment of differentiation was performed using AdipoRed^®^ under a fluorescence microscope (Fig. 5A–F). The dye is a fluorescent marker for intracellular lipid accumulation. Semi-quantitation was performed assessing the differential fluorescence intensity in ASCs derived adipocytes on NW with or without exposure to MF compared to controls treated with maintenance culture media using Image J software (Fig. 5G). Quantitative evaluation of lipid accumulation was performed using spectrophotometry and results expressed relatively to DNA content and to ASCs differentiation cultured in similar conditions. We found that ASCs cultured on NW substrate retained their adipogenic potential, displaying however significant decreased lipid accumulation compared to controls in regular culture plates. MF exposure significantly reduced adipogenic ability of ASCs cultured on both regular substrates and NW substrates (Fig. 5H).

### ASCs differentiation on NW substrate—osteogenesis

**Figure 6:**
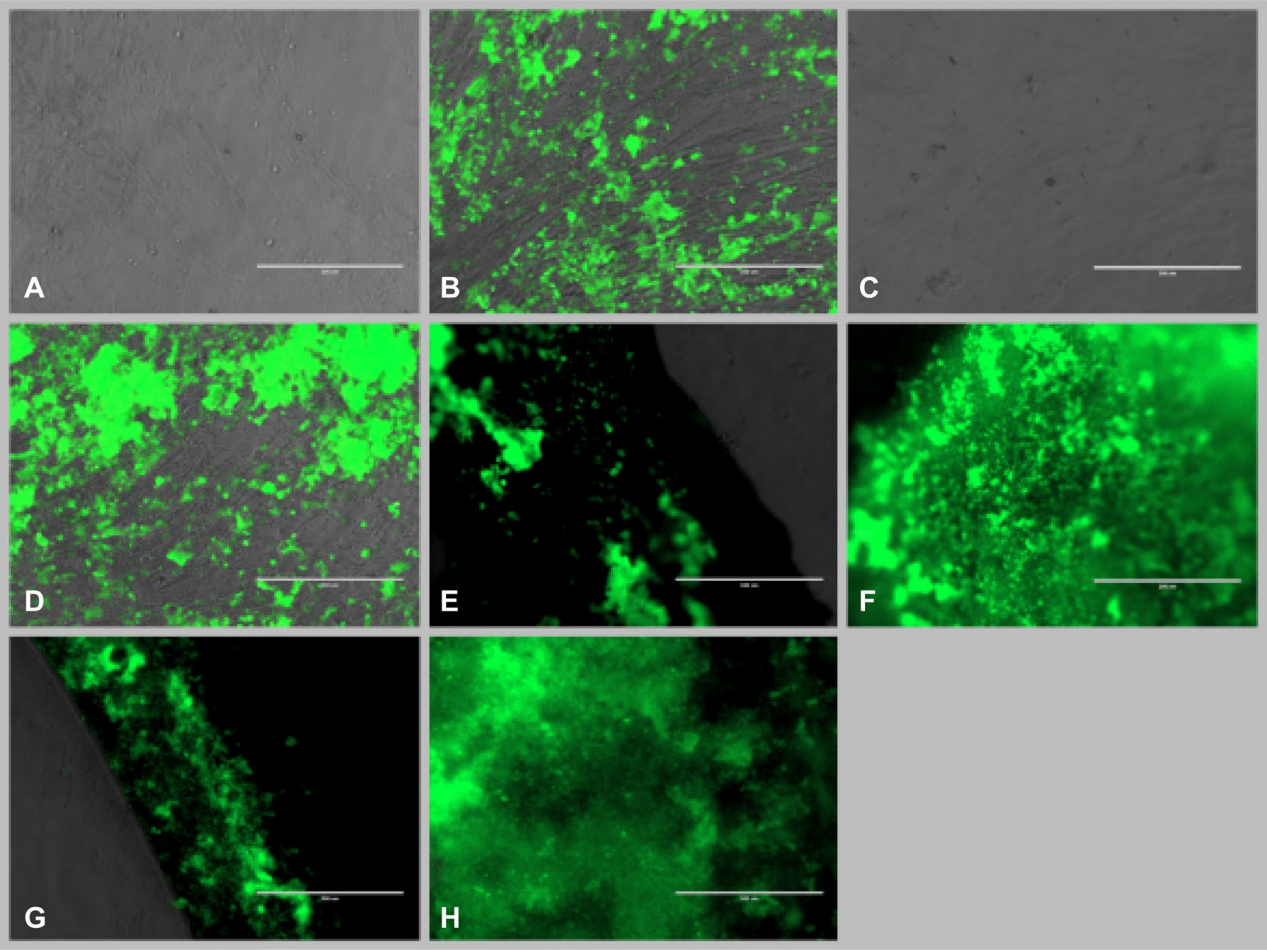
ASCs differentiation: (**A–H)** fluorescence microscope images of osteogenic differentiation: (**A**) control (ASCs on regular culture plates in simple culture media); (**B**) ASCs in regular plates in osteogenic media; (**C**) control MF exposed ASCs in simple culture media; (**D**) ASCs in regular plates in osteogenic culture media; (**E**) ASCs on NW substrate in simple culture media; (**F**) ASCs on NW substrate in osteogenic media (**G**); ASCs on NW substrates exposed to MF in simple culture media; (**H**) ASCs on NW substrates exposed to MF in osteogenic media OsteoImage^®^ fluorescent staining of mineralized matrix deposition (extracellular), all images represent overlay of transmitted and GFP fluorescence images captured with inverted fluorescent microscope (Evos FL Life Technologies).

**Figure 7:**
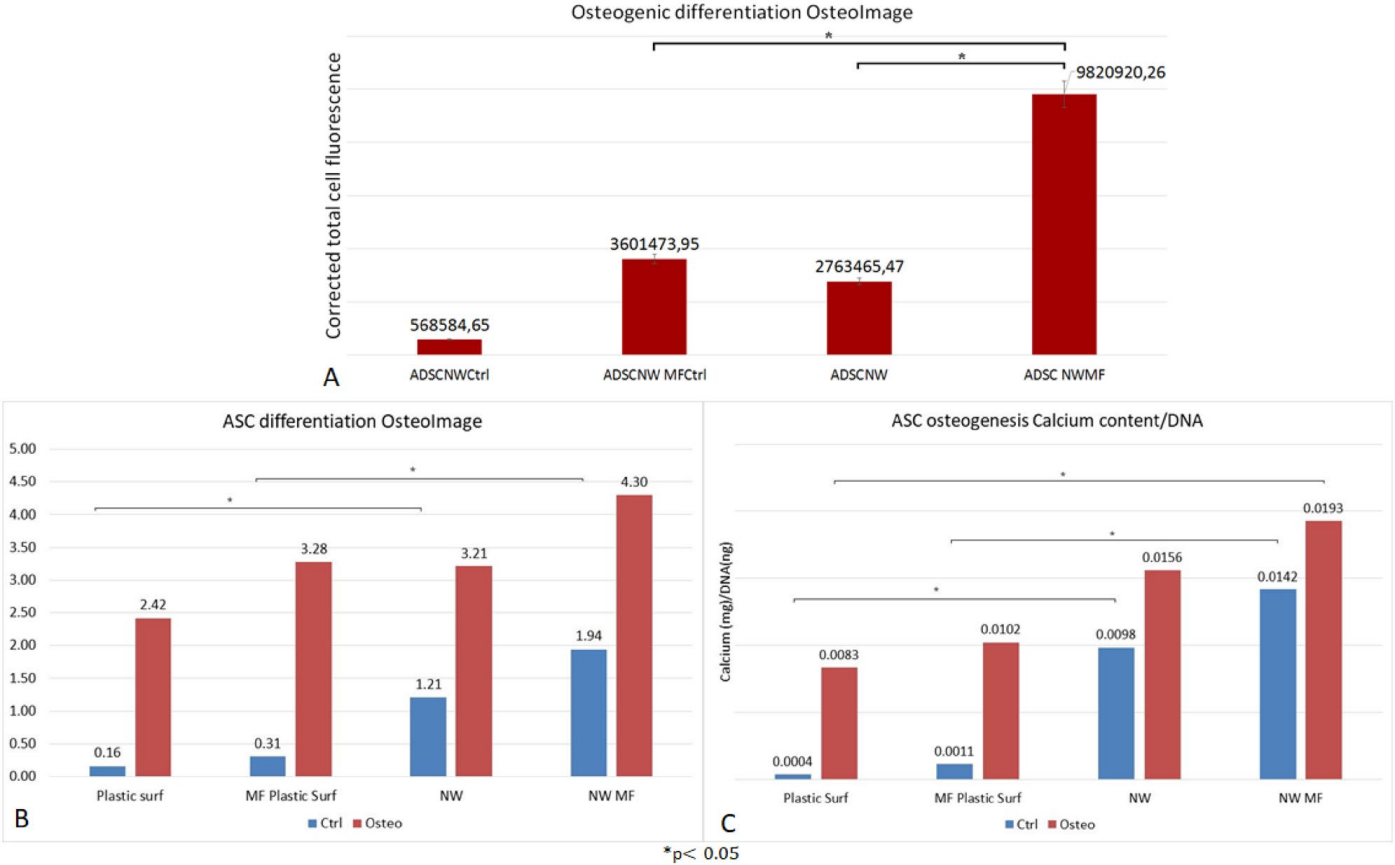
Quantitation of matrix mineralization expressing ASCs osteogenic differentiation: (**A**) semiquantitative assessment of media OsteoImage^®^ fluorescenxe using Image J software; (**B**) Comparative quantitative assessment of ASCs osteogenesis on regular adherent plastic surfaces and magnetic nanowire substrates (NW) exposed or non-exposed to 0,5 mT alternating magnetic field (MF) relative to DNA content; (**C**) assessment of Calcium content of the mineralized matrix deposited by ASCs on regular adherent plastic surfaces compared to NW surfaces exposed and non-exposed to MF.

For osteogenesis assays, seeding protocol, differentiation media and MF exposure as well as assessment of differentiation using OsteoImage^®^ was performed similarly with the adipogenesis protocol. The efficiency of osteogenesis was evaluated quantitatively based on the amount of ECM deposited by ASCs-derived osteoblasts by means of semi-quantitative evaluation of fluorescence using Image J as well as quantitative evaluation of ECM deposition revealed with OsteoImage using a plate reader. Quantitation of calcium content of the deposited ECM was used to confirm matrix mineralization and osteoblastogenesis. Remarkably, osteogenic conversion of ASCs cultured on NW substrate could be observed in ASCs treated with osteogenic media but as well as in control ASCs cultured in normal culture media exposed and non-exposed to MF. ASCs cultured on NW substrates treated with osteogenic media and exposed to MF resulted in significantly increased osteogenesis compared to non-exposed as well as to ASCs treated with normal culture media and exposed to MF. Increased mineralized matrix as well as calcium deposition was obtained from ASCs on NW compared to regular plastic surfaces, while significant increase was recorded from ASCs on NW substrates exposed and non-exposed to MF compared to their plastic surface correspondents (Fig. 6A–H). To note, maximum osteogenic conversion (as expressed by maximum fluorescence intensity normalized to DNA content as well as by calcium deposition normalized to DNA content) was obtained in ASCs treated with differentiation media and exposed to MF cultured on NW ASCs (Fig. 7A–C).

## Discussion

In this study, we tested the feasibility of using magnetic responsive NW as substrate for ASCs culture for the purpose of delivery of nano topographical cues as well as of substrate-induced mechanical stimulation to mesenchymal progenitors undergoing differentiation. In order to partially expose the tips of the electrodeposited Ni NWs array for the ASCs culture, we had to remove the remaining (non-filled part) of the Al_2_O_3_ membrane. For this purpose, the Al_2_O_3_ membrane was kept floating upside-down onto the surface of 3 M NaOH aqueous solution at room temperature for 8 min. We found the placement of NW pieces inside conventional culture dish to be facilitated by their maneuverability using magnetized handling instrumentation (forceps) and impaired by the fact that NW-filled membrane is rather brittle. For the purpose of translating to potential tissue engineering application, a method of obtaining larger similar substrates directly placed within bioreactors could be designed.

ASCs do not adhere preferentially to NW substrates unless placed inside ULA plates that do not present another adhesive alternative to cells in suspension after tripsinization. Previous studies reported a relatively low amount of human mesenchymal stem cells are able to adhere to NW substrates^17^. We tried to circumvent this inconvenient by placing NW inside ULA plates. We found that this simple variation of protocol can force primary ASCs used in this study to use NW surface as adhesive substrate, as confirmed by the abundance of cell nuclei detected in this case (Fig. 4G,H). The efficiency of cell seeding on NW can be regarded as satisfactory, retaining around 60% of cellular elements compared to plastic surfaces (data not presented, available on request) Conversely, cells only use the margin of the NW platforms when cultivated within adherent culture dishes (Fig. 4E,F). Cells display high viability on NW substrate for both A and ULA dishes; however, viability results were found to be method dependent. It has been reported that MTT assay can introduce errors when assessing the cytocompatibility of nanofibrous materials due to the step that involves cell lysis that can interfere with the material^18^. This can explain the differences between viability we obtained in the case of MTT compared to resazurin assay.

Qualitative investigation using LIVE/DEAD further confirmed good viability as well as maintenance of cellular enzymatic apparatus and population of the entire surface of the NW membrane for the case of ULA plates. Few cellular elements were visible, mainly on the edges of NW membranes, for the case of adherent plates (Fig. 4A,B). Indeed, there is evidence of good viability of human mesenchymal adult and progenitors of various tissue of origin in contact with iron-core NW^19,20^ as well as for Ni based nanowires^21^. Higher concentration (11.88 μg/ml) and more than 24 h exposure to Ni NW were reported to induce inflammation of endoplasmic reticulum in human fibroblasts in the case or their internalization by the cells^22^. In this study Ni NW, non-detached from their alumina membrane, are used as substrate by ASCs that normally display an adherent phenotype, possible as a modality to avoid anoikis. Anoikis, a form of programmed cell death, is known to occur due to the loss of anchorage-dependent attachment to the extracellular matrix (ECM)^23^. A low propensity to adhere, due to a loss of matrix and intercellular anchorage, may induce the death of transplanted MSC^24^. This can possibly explain the low number of cellular elements captured on NW substrate in the case when cells were presented the opportunity to adhere to normal culture dish left unoccupied by the NW platform.

In the case of ASCs placed in adherent plates, very few cellular bodies with rounded shape or ruffled membrane could be detected by SEM imaging while in the case of ULA plates, cells retain their fibroblastic appearance, forming the intertwined network usually seen for mesenchymal cells on 2D substrates (Fig. 4C,D).

Cytoskeleton appearance in ASCs further confirms this observation. Phalloidin-stained actin fibers occupied the whole surface of NW platforms in ULA plates (Fig. 3D,E) as opposed to insular appearance and scarce, rather marginal, disposition of ASCs elements and cytoskeleton in cells on NW substrates in adherent plates (Fig. 3A,B). As compared to cytoskeletal distribution of ASCs in regular plastic adherent plates cells layered on NW in ULA plates seem to display a rather intertwined configuration that could be detected as well on SEM (Fig. 1G) with less intercellular distances and closer cell to cell contact. This can be explained from one side by the fact cells continued to proliferate on a restricted surface area. Another possible explanation could be the presence of the relative uniform substrate-induced nano topography. Here, NW surface available for cell adherence was uneven with protruding pillars displaying 0.1–1 µm differences between their tips (Fig. 1B). Complex cell shape and intercellular body connection in fibroblastic cells was previously reported on microscale pillars. Cells were found to spread less on higher silicon pillars (up to 10 µm) and possess similar configuration to planar substrates on pillars less than 1 µm^25^. Elsewhere, decreased expression of integrin subunits (α2, α6, αV, β2, β3 and β4) were reported in human MSC on 350 nm gratings (nanogroove topography) of tissue culture polystyrene (TCPS) and polydimethylsiloxane (PDMS), compared to the planar control^26^. Specifically, the integrin “adhesome” was proposed to induce structural and signaling protein recruitment result in formation of larger adhesion structures (adhesion maturation)^27^. Focal adhesion (FK) comprised of integrins, structural proteins, adaptor proteins, signaling molecules, and cytoskeletal component govern intercellular connectivity and cell alignment on nontopographical substrates^28^. Vinculin, as a marker for FK was previously shown to “concentrate” around pillars in human MSC cultured on NW however in that study the NW endings were shown to come together in bunches^17^. The described ”flower-like” configuration of the substrate that presents larger gaps^17^ is obtained when the alumina membrane is dissolved completely (Fig. 1C). In our study, we chose to provide the cells a uniform substrate by dissolving the alumina membrane only in part (Fig. 1B). The compactness and alignment of cellular bodies as well as of cytoskeleton actin fibers could result from FK particularities displayed by cells attaching on uneven 1–2 µm scaled but parallel pillars, fact that needs further confirmation. Previous study revealed that human MSC cultured on vertically aligned silicon nanowire (Si NW) displayed good viability and comparable proliferation compared to ones cultured on regular culture dish^29^. Due to technical limitations, we have not performed here a quantitative comparative evaluation of the number of cells attached to NW substrate in the case of NW placed in ULA plates, and regular adherent plastic culture dish. However, qualitative information resulting from observing the amount of cell nuclei on NW placed in adherent and ULA culture dishes lead to the conclusion that very few cells can be found in the first situation. We decided to perform differentiation experiments with cells on NW in ULA plates only. Since the purpose of the study was to investigate if ASCs differentiation on NW is influenced by MF exposure, a comparative investigation of the conversion efficiency between NW substrates and regular adherent plastic dishes was performed as well.

Considering placing NW-filled alumina membrane within alternating MF for delivery of magneto-mechanical stimulation, we performed a theoretical modelling^30^ for approximating the magnetic field distribution, especially around the ends of the nanowires. The dynamic of the NW in the alternating MF produced by the coils depends on a series of factors. First, because NW are outside the membrane at their top (Fig. 1A), the free endings follow the dynamic processes imposed by the MF and bend depending on the intensity and frequency of the applied field. This leads to a complex fluctuation of the field in the entire cells area, while allowing the fields generated around the tips to pulsate inside the cell with the frequency of changing the polarity of the coils. The bending amplitude and the associated radius of curvature are related to a series of forces such as magnetic, inertial, elastic, and gravitational, friction or even Colombian, given the short distance between nanowires (Fig. 1B) and the processes of friction resulting during the bending. These forces are depending on composition, size and shape of the nanowires, their specific magnetic properties (such as magnetization, remanence or coercivity), magnetic/electrical interactions related to the distance between the free ends of the nanowires, temperature (which may increase locally when the nanowires rub among them), the physical properties of the environment (e.g., viscosity, magnetic permeability, and electrical permittivity of the cell culture medium). Also, the influence of increasingly acidic pH in cell culture media may affect the NW magnetic material in time, including the integrity of the NW surface, fact that should be taken into consideration and quantified when longer exposure times and/or upscale manufacturing are eventually envisaged.

It should also be noted that during MF application, in addition to the tensile forces, shear forces on the cell membrane may be possible. Since the cells are attached to NW endings, these forces can have a strong influence on the dynamic and geometry of the whole cell with a maximum effect at its inferior pole, especially at the NW-membrane interface. Therefore, in order to keep such effects at minimum level, we used a low-intensity field with a very low frequency to allow the nanowires to slowly follow the variation of the magnetic field.

Here we found that MF exposure does not favor adipogenesis of ASCs on magnetic NW, while adipogenesis was found to be significantly higher in ASCs non-exposed to MF. Evaluation of osteogenesis and adipogenesis, respectively, was performed quantitatively investigating the amount of ECM deposited by differentiating cells or lipid content respectively, reflecting efficiency of differentiation in terms of functional protein expression. Previous reports point towards the negative influence of oscillating MF and mechanical vibration on MSC adipogenesis. Our group has previously reported that intermittent MF exposure significantly decreases adipogenesis of ASCs loaded with as-prepared Fe_3_O_4_ nanoparticles (MNP), but as well in non-loaded controls^31^. Oscillating MF exposure and mechanical vibration were found to induce cytoskeleton remodeling resulting in F-actin fiber rearrangement and consecutive significant decrease in gene expression of adipogenesis master regulator peroxisome proliferator-activated receptor gamma (PPARγ), adiponectin and adipocyte protein 2 (AP2)^32^. Adipogenic activation further disturbs cellular actin stress fibers by down regulating RhoA-ROCK signaling pathway^33^. Actin stress fibers have been reported to be strongly affected by both static and alternating MF exposure, pointing towards a sustained negative influence of MF exposure in the days following initiation of differentiation. Here we found that while adipogenesis on NW substrates is still possible, the efficiency of adipogenic conversion is significantly lower compared to plastic surfaces and further decreased by MF exposure.

Significant increase in osteogenic conversion was observed in this study in ASCs on magnetic NW exposed intermittently to low intensity MF for 7 days after initiation of differentiation compared to non-exposed (Fig. 7A–C). What was even more remarkable was the fact that ASCs on NW treated with normal culture media and exposed to MF displayed comparable osteogenesis with counterparts treated with osteogenic media, non-exposed to MF and particularly to the plastic adherent cultured counterparts. This is important since NW substrate exposed to MF were proven able to induce osteogenesis in ASCs even without the presence of biochemical cues for triggering conversion. Previous reports on mouse bone marrow MSC cultured on 9-15 μm long, vertically aligned, silicon NW were shown to preferentially undergo osteogenesis and chondrogenesis, but not adipogenesis in the absence of supplementary growth factors. Authors reported that stretch mediated Ca^2+^ ion channels were transiently activated in MSC upon topographical induced mechanical stimulation, leading to activation of Ras/Raf/MEK/ERK signaling cascades that could eventually contributed to upregulation of osteogenic and chondrogenic gene expression and downregulation of adipogenic regulators^33^. Here, the endings of the NW did not clump together in a spike-like manner, while forming larger gaps in between in the manner reported in other studies^17,29^, but rather presented a homogenous substrate with relatively repetitive unevenness. This effect was obtained by tailoring NW fabrication by removing only in part the aluminum membrane in order to improve NW surface configuration designed for cell attachment. Some of the forces involved in fluid circulation might as well affect the transfer of fluid/nutrient through the plasma membrane, especially in the region where the cell is anchored on nanowires. This transfer may also be governed, inter alia, by capillary forces and/or surface tension rising between high-density nanowires, and the interface between the nanowires and the cell membrane.

In this study, MF exposure as well as NW-related induced magneto-mechanical stimulation contributed to osteogenic conversion even in the absence of external biochemical cues. Previous reports on MSC of various origin point towards a causal correlation between electric fields (EF)^34^ as well as MF exposure and osteogenesis. In human dental pulp stem cells (DPSC), 1mT static MF exposure had as an effect the recruitment of YAP/TAZ (Yes-associated protein / transcriptional coactivator with PDZ-binding motif) to the nucleus, inhibition of YAP/TAZ phosphorylation and upregulation of downstream genes, connective tissue growth factor (CTGF) and ankyrin repeat domain-containing protein (ANKRD1).Both cytoskeletal inhibition with cytochalasin D as well as YAP/TAZ knock-down, abolished mineralization in MF. YAP/TAZ the main effector in hippo pathway cross talk with cytoskeleton to mediate MF induced osteogenesis^35^. Importantly, not only bone marrow MSC, but as well ASCs, notoriously less prone to undergo osteogenesis^36^, are able to increase matrix mineralization under pulsed electromagnetic field (PEMF) exposure, supporting their use in for bone tissue engineering^37^. To note, PEMF are already used in clinic for accelerating bone healing during complex fractures or non-unions regardless the fact that biological processes backing this effect have not been precisely described^38^. This effect seems to be further increased by the presence of magnetic responsive nanomaterials. ASCs loaded with MNPs exposed to alternating MF or stimulated by MNPs within static MF were shown to increase osteogenesis^30^ or to mediate pro-osteogenic and pro-angiogenic activity respectively by means of the release of exosomal miR-1260a^39^.

Given that the average thickness of the cell and its membrane is about 5 μm^40^ and 10 nm, respectively, depending on cell type and/or physiological processes, the strongest influence of the magnetic field could be produced on the cell membrane and the endoplasmic reticulum as well as on the iron-rich cell endoplasm in itself. The cell membrane is a major target for alternating magnetic fields (AMF), electromagnetic fields (EMF) because they can modulate ligand binding and ion fluxes involved in human bone marrow MSC osteogenic and chondrogenic differentiation^41^. This effect can potentially be enhanced by the microfluidic flow of media in between NW that can act like a suction capillary network, fact that needs further confirmation.

While majority of the studies support increased osteogenesis in MSC of various origin under MF exposure^41–43^, earlier reports argue about inhibition of cell growth and metabolic features even when differentiation efficiency remains comparable to non-exposed cells^42^. Results from various reports differ depending on the experimental and environmental conditions especially related to MF characteristics’ parameters (such as frequency, intensity, and time of exposure) are directly correlated with the efficiency of in vitro differentiation in MSC^41^. Therefore, they need to be adjusted accordingly.

Here, we used theoretical modelling of NW endings that induce magneto-mechanical effect as well as our previous results regarding timing and duration of exposure in order to establish MF exposure protocol. It is presumable that this protocol needs to be readapted for the case such substrates could be manufactured to occupy larger surfaces (for example in prefabricated dishes or bioreactor shelves). Magnetic Ni NW could be used as platforms for inducing MSC differentiation even without exposure to biochemical cues. This would consistently simplify manufacturing protocols for bone engineering application. Surface modification combined with MF exposure could be used as a modality to enhance Ni NW coated orthopedic implant osteointegration while preventing adipogenicity of local recruited progenitors. Further in vivo studies are mandatory for confirming the validity of these findings in the complex bone forming or bone repair niche where the role of local and circulating immune components need to be assessed.

## Conclusion

In this study, we have reported for the first time, as to our knowledge, superior osteogenic, but not adipogenic differentiation of adipose-derived stem cells on Ni NW exposed to alternating magnetic fields and compared to regular plastic adherent surfaces. Moreover, magnetic actuation of the magnetic nanowires was shown to induce osteogenesis, but not adipogenesis, even in the absence of mandatory biochemical stimuli regularly used for MSC differentiation.

The results showed that ASCs morphology and cytoskeleton appearance, when cultured on nickel nanowires (NW) substrate, differ consistently when the substrate is placed within non adherent culture dish compared to regular culture dish. Cells retain fibroblastic appearance and form dense cellular networks that appear to be favorable for their viability on these substrates.

Using a modified protocol for fabrication of Ni NW substrates, a significant increase in the osteogenesis of ASCs cultured on magnetic NW substrate exposed to alternating MF compared to non-exposed was observed. Adipogenesic conversion in similar conditions was found to be significantly decreased. Remarkably, ASCs underwent osteogenic conversion, but not adipogenesis on NW substrate under MF exposure in the absence of specific differentiation media.

The theoretical simulation showed a potential strong influence of pulsating magnetic fields on the plasma membrane at the anchor points with NW’s tips. Also, the analysis showed an uneven distribution of magnetic fields both horizontally and vertically in the immediate vicinity of the NW ends. The increased alternating magnetic field gradients in both directions may greatly influence the ion fluxes, especially the voltage-gated pores of the plasma cells.

The results showed that Ni NW substrates subjected to alternating MF could be used for bone tissue engineering application. While these findings need to be validated in vivo, we assume that orthopedic and/or dental implants surface modified with matrix of nickel nanowires subjected to extremely low-frequency magnetic fields could have a fine potential to improve osteointegration and implant stability.

## Material and methods

### Magnetic nanowires

Magnetic Ni NW was synthesized by electrodeposition method into commercially available porous alumina (Al_2_O_3_) templates from Whatman Inc. We used Anodisc 25 membranes with a thickness of 60 µm (i.e., the maximum length of the NWs which could be deposited), pores diameters of 200 nm, and pore to pore distance of 200–250 nm. The average pores density was 109 cm^−2^ and surface porosity of 25–30%. Before electrodeposition, a 250 nm Cu thin film was sputter deposited onto the backside of the Al_2_O_3_ template. The copper back-electrode played the role of the working electrode, a saturated calomel electrode (SCE) was used as the reference electrode, while the counter electrode was a platinum wire. We used a parallel arrangement, with a distance between the working and counter electrode of about 50 mm. Electrodeposition experiments were carried out in controlled potential mode using different pulse durations and voltages in order to tune the length of the as-deposited Ni NW.

The electrochemical bath for Ni NW electrodeposition was a sulfate-based aqueous solution containing boric acid (H_3_BO_3_), ammonium chloride (NH_4_Cl), nickel sulfate hexahydrate, sodium saccharin, and sodium dodecyl sulfate. The exact composition in g/L of the electrolyte solution used for Ni NW electrodeposition is given in Table 1.

**Table 1:** Composition and concentration of electrodeposition electrolyte.

Compound	Concentration (g/L)
H_3_BO_3_	24.73
NH_4_Cl	16.04
NiSO_4_·6H_2_O	105.12
Sodium saccharin	1
Sodium dodecyl sulfate	0.05

The applied pulsed potential consisted from pulse of 2.5 s at a “deposition “potential of − 1.15 V vs. SCE, followed by a 1 s pulse at a “rest” potential of − 0.7 V. This cycle of pulses was repeated as needed in order to reach the desired length of the Ni NW.

X-ray diffraction patterns of the synthesized NW were recorded using a Brucker AXS D8-Advance powder X-ray diffractometer (CuKa radiation, k = 0.1541 nm). Electron micrographs of the powders were taken by using a FIB/FE-SEM CrossBeam Carl Zeiss NEON 40 EsB equipped with an energy dispersive X-ray spectroscopy (EDS) module for compositional studies. The magnetization data were acquired on a Lake Shore 7410 vibrating sample magnetometer (VSM).

### Nanowire substrate for cell culture

The top part of the template with vertically protruding NW served as a substrate for cell culture. Rectangular pieces of alumina templates of 0.3/0.4 mm were washed twice with 96% ethanol and sterilized using an autoclave for 30 min at 121 °C and 2 × 10^5^ Pa. For viability and proliferation test, NW substrates were manually placed within 96 well plates using sterile tweezers. 96 well plates with flat bottom and adherent plastic surfaces as well as ultra-low adherent (ULA) 96 well plates were used comparatively (Sigma-Aldrich International GMBH). Prior to exposure to cell populations, NW substrates were washed 3 times with 100 µl complete culture media (Dulbecco modified culture media DMEM, 10% fetal bovine serum FBS and 2% antibiotic antimycotic). For adherent well plates, 10^4^ cells suspended in 70 µl CCM are added to NW drop by drop and placed within the incubator overnight. 130 µl medial is added after 7–8 h to prevent cell drying. For ULA well plates, 10^4^ cells suspended in 150 µl CCM were added on the top of wells containing NW.

### Theoretical simulation of the magnetic fields in the nanowire-cell region

To analyze the magnetic field around the tips of magnetic nanowires, we used a finite element package (Finite Element Method Magnetics—FEMM ver. 4.2) for solving 2D planar and axisymmetric problems in low frequency magnetics and electrostatics.

### Human primary adipose derived mesenchymal cells

Human primary ASCs were obtained from healthy donors undergoing elective cosmetic liposuction procedures after institutional ethical approval and patient consent. The resulting lipoaspirate was processed within a maximum of 24 h after the surgical procedure (average of 2 h) as previously described^44^. Briefly, lipoaspirate was washed with PBS, digested with collagenase type I (0.01 mg/ml) for 2 h at 37.5 °C, and centrifuged at 300*g* for 5 min at room temperature. The supernatant was removed and the medium was further centrifuged at 300*g* for 5 min. Pelleted cells were re-suspended in complete culture media Cells, automatically counted (TC20™ Automated Cell Counter, Bio-Rad Hungary Ltd), and plated in appropriate tissue culture flasks (CellBIND surface, Corning). ASCs from passages 2–4 were used for experiments. For morphology evaluation, the cells were observed under a fluorescent inverted microscope (EVOS Fl Life Technologies). Cell phenotype was characterized in terms of surface markers and mesenchymal lineage differentiation (Supplementary material).

We declare that all experimental protocols were done in accordance with relevant European guidelines and regulations, following the approval of protocols by the Ethical Committee of County Emergency Hospital Saint Spiridon Iasi Romania No 31567/1507/2015 and Ethical Committee of County Emergency Hospital Saint Spiridon Iasi No 23/01/08/201.

### ASCs morphology on magnetic nanowires

Pieces of alumina membranes, previously placed in adherent and ULA 96 well plates and seeded with ASCs, were observed using an inverted fluorescent microscope as well as scanning electron microscope (SEM). For SEM imaging, samples were washed with PBS, cells were fixed with glutaraldehyde solution (2.5% in cacodylate buffer) for 2 h at room temperature. The pieces were stained in 1% osmium tetroxide (in cacodylate buffer) for 1 h in dark. Samples were dehydrated in graded series of ethanol (10–100%). In the final stage, the pieces were coated with Au and imaged using SEM.

### ASCs viability on magnetic NW

For cytotoxicity assays, ASCs were plated in adherent and ULA 96 well plates, at 2 × 10^4^ cells/well and incubated for 48 h. Cell viability tests were performed using MTT (5-dimethylthiazol-2-yl-2, 5-diphenyltetrazolium bromide—Vibrant^®^ Thermo Fisher Scientific) as well as resazurin assay (Resazurin sodium salt, Sigma Aldrich) according to supplier’s instructions. Briefly, for MTT, dimethyl sulfoxide (DMSO) was used as dilution agent and absorbance was read at 570 nm using a plate reader (Synergy HTX Multi-Mode Reader- Biotech). For the resazurin assay, 20 μl of resazurin solution was added to each well and incubated for 24 h. The absorbance was read at 595 and 570 nm. Cell viability (CV), expressed by MTT optical density (OD), was calculated using the formula CV = 100 × (ODs-ODb)/(ODc-ODb), where ODs = OD of particle treated cells; ODb = OD of blank (media only); ODc = OD of untreated cells.

### Assessment of ASCs and cytoskeleton fibres on NW

2 × 10^5^ cells (passage 3–5) were plated in 35 mm Petri dishes CCM cells were fixed with 2% paraformaldehyde for 15 min, stained with phalloidin (Texas Red™-X Phalloidin, Termo Fischer Scientific) and counterstained with nuclear staining with 4′,6-diamidino-2-phenylindole (DAPI). Images were taken using a fluorescent inverted microscope (EVOS Life Technologies).

### ASCs differentiation on magnetic nanowires

ASCs (2 × 10^4^) seeded on NW plates were inserted within ULA 96 well plates in complete culture media (CCM). After 24 h, CCM was washed with PBS and replaced with osteogenic (single Quots™ osteogenic media Lonza) or with adipogenic (single Quotes™ adipogenic induction and maintenance media Lonza) for osteogenesis or adipogenesis assays respectively, fed each 3 days for 21 days (osteogenesis) or until completing three cycles of induction-maintenance for adipogenesis respectively. Cells were fixed with 98% ethanol for 10 min washed with PBS.

#### Assessment of differentiation—osteogenesis

Osteoimage™ osteogenesis assay (Lonza, Walkersville US) was used for qualitative and quantitative assessment of osteogenesis, accordingly to the provider’s instructions. Briefly, fixed cells were washed with diluted wash buffer (WB), then 0.5 ml/well of diluted staining reagent was added, followed by plate incubation for 30 min at room temperature (RT). After washing three times with WB, the samples were observed with an inverted fluorescent microscope (Evos FL Life Technologies). For semi-quantitative osteogenesis assay, at least 5 images per field of view were recorded and fluorescence was quantified as maximum fluorescence intensity using Image J software. The areas of interest were delimited and measured, as well as the area with no fluorescence for background. The data resulted was selected and integrated into the following formula to calculate the corrected total cell fluorescence (CTCF). CTCF = Integrated density − (Area of selected cell * Mean fluorescence of background readings). This step was repeated for all the recorded images. For quantitative osteogenesis assay fluorescence was read at 492/520 excitation/emission using a plate reader. For assessing calcium content cells after fixation with 90% alcohol assays were treated with 0.5 mM HCl. Calcium content per well was assessed using Calcium LiquiColor (EKF Diagnostics Inc. USA) following provider instructions. Results were quantitated using absorbance values obtained using a plate reader plotted against a standard curve for calcium content (mg). Differentiation assays results were normalized to DNA content The results were normalized for total DNA obtained using Quant-iT Pico Green dsDNA assay (Invitrogen) as per manufacturer instructions.

#### Assessment of differentiation—adipogenesis

Adipogenesis was quantified using AdipoRed™ assay (Lonza, Walkersville, US) accordingly to manufacturer’s instructions. Briefly, the cells were double washed with PBS, and AdipoRed was added to wells. After short incubation assays were imaged using an inverted fluorescent microscope. At least 5 images were recorded per field of view. Semi quantitation of fluorescence was performed using ImageJ software as described for osteogenesis. For quantitative assessment of lipid content, AdipoRed fluorescence was read in the plate reader at 485 nm excitation and 524 nm emission. Results were normalized to DNA content as described for osteogenesis.

#### ASCs differentiation on magnetic NW under MF

Differentiation assays were performed under exposure to MF (4 mT) for 10 min every 2 h during the first 7 days after initiation of differentiation (represented by the first media change from expansion to differentiation media). One ULA 96 well plate unexposed to MF was kept in the CO_2_ incubator. Regular adherent 96 well plate without NW inserts were used as controls for ASCs differentiation.

For magnetic field generation, a conventional Helmholtz system consisting of two coils was used. The proprietary software that controlled the coils allows to set the magnetic field intensity, its frequency and exposure time. The coil system was fed by waveforms generated using a code realized in LabView. The culture plates were placed in the center of the coil system, where the magnetic field is uniform. The magnetic field was shifted by 180 degrees with a frequency of 1 Hz. The system can generate fields of 1–40 Oe (Fig. 2C).

### Statistical analysis

All experiments were performed in biological triplicate. One way analysis of variance (ANOVA) was performed using OriginLab PRO version 8.0) followed by Bonferroni post hoc analysis; between-group and within group differences that were considered significant at p < 0.05 (n = 3).

## Supplementary Information

Supplementary Information.

## Data Availability

The datasets used and/or analysed during the current study available from the corresponding author on reasonable request.
