# Supplementary material for: Magnetic nanowires substrate increases adipose-derived mesenchymal cells osteogenesis
Source: Sci Rep. 2022 Oct 6;12:16698. doi: 10.1038/s41598-022-21145-z (PMC9537172; doi:10.1038/s41598-022-21145-z)
Supplement: Supplementary file 1 — Supplementary Information. [file 41598_2022_21145_MOESM1_ESM.docx]

Assessment of mesenchymal stem cell phenotype (Dominici et al 2006)

1.All cells were cultured in 2D except chondrogenic pellets and were proved to be adherent to the polystyrene of tissue culture dishes.

2.Surface markers analysis was performed using a Guava flow cytometer (Guava Easycyte, Luminex Corporation) for thawed cells in P2. Antibodies FITC/PE antibodies for CD105 (Invitrogen) CD 73 and 90 (BD Biosciences), were used as positive surface markers of mesenchymal stem cell identity; CD 34 CD 45 (BD biosciences), negative surface markers, HLA-Dr (Invitrogen), γ1, γ2a (BD Biosciences), negative controls γ12b (BD Biosciences) isotype control

Supplm. chart 1. Percent of positive adipose derived cells for respective cluster of differentiation (CD) antigens within the gated population. Representative results from one donor

ASCs underwent trilineage differentiation (osteogenesis, adipogenesis, chondrogenesis) - Osteogenesis and adipogenesis are presented within manuscript text as regular plastic surface -grown ASCs)

For *chondrogenesis assays* 9x10*5 ASCs were pelleted in incomplete chondrogenic media (ICM) composed of - DMEM (high glucose -HG), Dexamethasone 1mM, Ascorbic acid 2-P: 5mg/ml L-Proline: 4mg/ml ITS+ supplement Sodium pyruvate). After pelleting, ICM was changed with complete chondrogenic media (ICM plus TGFβ-3 10ng/ml). Chondrogenic pellets were kept in 15 ml polypropylene tubes in incubator and fed twice/week for 21 days. Histological evaluation was performed after fixing with PFA 20% solution for 24 h.

Figure 1. Supplementary material Histological evaluation of representative chondrogenic pellets (Safranin O staining)


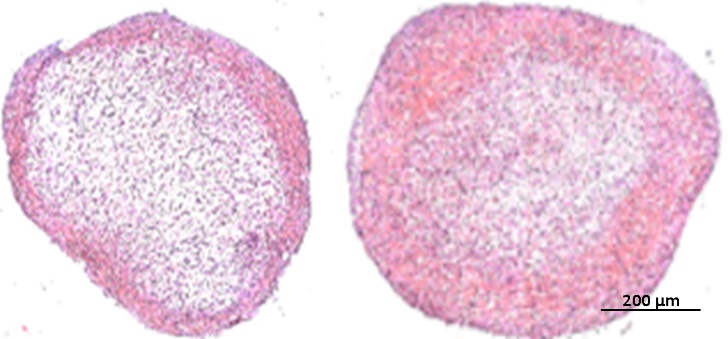


Supplm. Chart 2 Comparative cell number ASC cultured on plastic adherent and NW substrates
